# The Future of Anaesthetic Pharmacology

**Published:** 2009-10

**Authors:** Y K Batra

**Affiliations:** Professor, Department of Anaesthesia and Intensive Care, Postgraduate Institute of Medical Education and Research, Chandigarh, India

Anaesthetic practice is unique unlike other branches of clinical medicine requiring rapid onset and offset of pharmacological action. Anaesthesia is a dynamic state of the brain with wide variations and fluctuations, requiring continuous adjustment of drug dosing. Maintenance of the fine balance between the antagonistic forces of drug dosage and stimulus applied thereby ensuring adequate depth is a clinical challenge. The profound physiologic alterations of the anaesthetized state (and their reversal) to be produced on demand makes anaesthesiologists to increasingly rely on drugs with rapid onset and predictable offset of effect. The future of anaesthetic pharmacology will be towards the development of drugs and routes of administration with faster and predictable effects with easy reversibility of action and lesser side effect profiles.

## Do we need a new hypnotic – sedative agent?

The development of an ultra-short acting hypnotic agent without undesirable effects is an unfulfilled dream. Apart from a search for newer drugs, removal of undesired effects while maintaining their beneficial effects is being tried for many drugs formerly withdrawn. Resurgence of previously withdrawn drugs like etomidate and propanidid, with better safety profile may soon replace the currently popular drugs like propofol. Probable candidate drugs include etomidate-like molecule, methoxycarbonyl-etomidate (MOC-etomidate)^1^, a rapidly acting, propranidid relative THRX-918661^2^, CNS-7056^3^, an esterase metabolized, short-acting benzodiazepine and melatonin analogues^4^. MOC-etomidate preserves etomidate's desirable hemodynamic profile but eliminate the suppression of adrenal corticosteroid synthesis associated with etomidate. It undergoes rapid metabolism by esterases thus teminating its hypnotic action. Melatonin and melatonin analogs possess hypnotic properties when injected intravenously, comparable to the properties of propofol and thiopental including the EEG effects with the additional advantage of providing analgesia, at least in animal studies. They may be candidates for being complete anaesthetics in providing both hypnosis and analgesia.

Water soluble formulations of propofol are being developed to address the disadvantages of currently available lipid formulations like bacterial growth, pain on injection, hyperlipidemia with resulting acidosis and organ dysfunction. Fospropofol (Aquavan)^5^, a water-soluble, phosphate-linked propofol prodrug is one of the new sedative drugs recently approved for clinical use, which upon hydrolysis releases phosphate and formaldehyde. The propofol concentration peaks approximately 8 minutes after an injection of making it unsuitable as an anaesthetic induction agent.

Similar drugs include water soluble propofol polymeric micelles formulation^6^, Propofol/ethanol/water formulation^7^, propofol – cyclodextrin combinations^8^ which have similar pharmacokinetics as the lipid formulations in animal models and soon may replace the latter as inducing agents. Ethyl dioxy phosphate prodrug of propofol^9^, a safer alternative for phosphonooxymethyl prodrug (fospropofol) releases acetaldehyde, a less toxic compound than the formaldehyde released from fospropofol is in developement.

Newer benzodiazepine receptor agonists like JM-1232^10^, which also has the property of lowering shivering threshold apart from sedative action may find a place in inducing therapeutic hypothermia in future.

## Future of inhaled anaesthetics-injectables or nobles?

Isoflurane had been given intravenously as a lipid emulsion to induce general anaesthesia in mice as soon as in 1995 and has been compared to propofol regarding the time course of action in animal studies. Soon, the lipid formulations may enable administering volatile agents during the cardiopulmonary bypass (CPB) when the lungs are excluded^11^.

Xenon,^12^ the noble gas with hypnotic and analgesic properties and may soon be available for commercial use replacing nitrous oxide in the near future. Helium^12^, another noble gas although devoid of anaesthetic properties has demonstrated organ protection in ischemia – reperfusion situations and may serve an adjunct to anaesthesia in future.

## Taking out the fear of the neuromuscular blockers

Gantacurium^13^, a new class of nondepolarizing neuromuscular blockers called asymmetric mixed – onium chlorofumerates was developed to replace suxamethonium. The structural resemblance to mivacurium with metabolism by alkaline hydrolysis in plasma and spontaneous formation of cysteine adducts, thus deactivating the molecule makes it very less susceptible to genetic variability in the population. Gantacurium comes closest to a true succinylcholine replacement of any nondepolarizing muscle relaxant to date.

A study in human volunteers showed that at 0.18mg.kg^-1^ time to onset of 90% blockade was 2.1 ± 0.6 min. and increasing the dose to 0.36mg.kg^-1^ shortened the onset to 1.3 ±0.2 min. The duration ranges from 4.7 to 10.1 min and spontaneous recovery occurs within 12-15 min after administration of as large as 0.54 mg.kg^-1^. Transient cardiovascular effects were observed at higher doses and were suggestive of histamine release.

AV002^14^ is another non-depoarising neuromuscular drug of intermediate duration and was designed to undergo cysteine adduction and possibly chemical hydrolysis more slowly than gentacurium. In dogs, administration of 0.08 mg/kg of AV002 resulted in duration of action of 71±4 min. The residual neuromuscular effects could be antagonised with neostigmine.

Among drugs reversing the neuromuscular blockade, sugammadex^15^, a cyclodextrin specifically designed to bind rocuronium thereby rapidly and completely reversing neuromuscular blockade is currently available in European union. In human studies, sugammadex, 8.0 mg.kg^-1^, reversed neuromuscular blockade within 1 minute of administration, without any apparent toxicity.

The future is going to see development of more short acting nondepolarising muscle relaxants with early onset of action, rapidly replacing succinylcholine, and selective relaxant binding agents (SRBA's) for the available neuromuscular blockers thus making neuromuscular blockade safer and reversibility of drug action more predictable. The development of such drugs may make postoperative residual paralysis a thing of the past.

## Recent trends in pain pharmacology

Future pain physicians may soon find administration of opioids safer with a wide variety of routes. Selective antagonism of the opioids overdose without compromising for analgesia will soon be a realistic goal. Alvimpan and methylnaltrexone, two peripherally acting opioid antagonists are orally delivered drugs approved for preventing opioid-induced ileus^16^. Recently, a 5HT4 (a) agonist, BIMU8, selectively reversed fentanyl-induced ventilatory depression, without affecting analgesic response in rats^17^. This creates the possibility that opioids could be coformulated with 5HT4 (a) agonists in future, preventing opioid induced ventilatory depression. Morphine-6-glucuronide^18^ the active metabolite of morphine although never introduced into clinical practice is currently being developed for post-operative pain. Initial studies suggest that morphine-6-glucuronide causes lesser ventilatory depression per an equipotent analgesic dose of morphine.

Routes of opioid administration are rapidly evolving with newer formulations and drug preparations. Recently, a single “DepoDur,” epidural morphine is approved in a liposomal formulation providing upto two days of effective analgesia^19^. Fentanyl apart from being available currently as transmucosal patch, lollypops and injectables is also being developed as sublingual fentanyl tablet called “Oravescent” providing more rapid onset than the oral transmucosal fentanyl delivery system, an inhaled form of free fentanyl having a rapid peak and offset, resembling intravenously administered fentanyl^20^. The rate of onset and the duration of effect can be modulated by encapsulating inhaled fentanyl in liposomes, an approach being explored. Durect pharma is developing a system to deliver systemic sufentanil over a period of months with an injectable osmotic pump intended for use in chronic pain^21^. Cannabinoids like ajulemic acid^22^, a novel cannabinoid with no psychotropic effects may be effective in chronic neuropathic pain in the future.

## Peripherally acting analgesics

Peripheral nociception often involves activation of the “transient receptor potential V1” (TRPV1) calcium channel. This channel, located mostly on C fibers in the periphery, is sensitive to capsaicin, acid, heat, and some lipids. Resiniferatoxin^23^ permits enough calcium to enter that the C fiber is permanently destroyed. Resiniferatoxin provides long-term analgesia making it a revolutionary drug for the treatment of severe chronic pain. Investigations have found topical application of antidepressants like amitriptaline and doxepin to be much more potent in antinociceptive effect and sodium channel blockade than local anaesthetics like bupivacaine^24^. They have been tried for topical skin application for cutaneous analgesia, mouth wash in oral cancer pain and may soon be alternatives for local anaesthetics with a better safety profile.

After a long hiatus, anaesthetic pharmacology is revolutionising towards development of drugs with safer and predictable effects with easy reversibility of drug action. The future is going to see developments in anaesthesia delivery systems, different routes of drug delivery and drugs having predictable pharmacokinetics with less dependency on genetic inter individual variability. The future anaesthesiologist will have an armamentarium of safe anaesthetic drugs with novel drug delivery systems and monitoring equipment making anaesthetic practice faster, simpler and safer.

## References

[1] Cotten JF, Husain SS, Forman SA, Miller KW, Kelly EW, Nguyen HH, Raines DE (2009). Methoxycarbonyl-etomidate: A novel rapidly metabolized and ultra-short acting etomidate analogue that does not produce prolonged adrenocortical suppression. Anesthesiology.

[2] Egan TD, Shafer SL, Jenkins TE, Beatie DT, Jaw-Tsai SS (2003). The pharmacokinetics and pharmacodynamics of THRX-918661, a novel sedative/hypnotic agent [abstract]. Anesthesiology.

[3] Kilpatrick GJ, McIntyre MS, Cox RF, Stafford JA, Pacofsky GJ, Lovell GG, Wiard RP, Feldman PL, Collins H, Waszczak BL, Tilbrook GS (2007). CNS 7056: A novel ultra-short-acting Benzodiazepine. Anesthesiology.

[4] Naguib M, Hammond DL, Schmid PG (2003). Pharmacological effects of intravenous melatonin: comparative studies with thiopental and propofol. Br J Anaesth.

[5] Gibiansky E, Struys MM, Gibiansky L (2005). AQUAVAN injection, a water-soluble prodrug of propofol, as a bolus injection: a phase I dose-escalation comparison with DIPRIVAN, part 1: pharmacokinetics. Anesthesiology.

[6] Ravenelle F, Vachon P, Rigby-Jones A E, Sneyd J R, Le Garrec D, Gori S, Lessard D, Smith D C (2008). Anaesthetic effects of propofol polymeric micelle: a novel water soluble propofol formulation. Br J Anaesth.

[7] Dutta S, Ebling WF (1998). Formulation-dependent brain and lung distribution kinetics of propofol in rats. Anesthesiology.

[8] Egan TD, Kern SE, Johnson KB, Pace NL (2003). The pharmacokinetics and pharmacodynamics of propofol in a modified cyclodextrin formulation (Captisol) versus propofol in a lipid formulation (Diprivan): an electroencephalographic and hemodynamic study in a porcine model. Anesth Analg.

[9] Kumpulainena H, Jarvinena T, Mannilaa A, Leppanena J, Nevalainena T, Mantylaa A, Vepsalainenb J, Rautioa J (2008). Synthesis, in vitro and in vivo characterization of novel ethyl dioxy phosphate prodrug of propofol. Eur J Pharmac Sci.

[10] Masamune T (2009). The shivering threshold in rabbits with JM – 1232(-), a new benzodiazepine receptor agonist. Int Anesth Res Soc.

[11] Rao Y, Wang YL, Zhang WS, Liu J (2008). Emulsified isoflurane produces cardiac protection after ischemia-reperfusion injury in rabbits. Anesth Analg.

[12] De hert S G, Preckel B, Schlack W S (2009). Update on inhalational anaesthetics. Curr Opin Anaesthesiol.

[13] Belmont MR, Lien CA, Tjan J (2004). Clinical pharmacology of GW280430A in humans. Anesthesiology.

[14] Naguib M, Bruil S (2009). Update on neuromuscular pharmacology. Curr Opin Anaesthesiol.

[15] Gijsenbergh F, Ramael S, Houwing N, van Iersel T (2005). First human exposure of Org 25969, a novel agent to reverse the action of rocuronium bromide. Anesthesiology.

[16] Taguchi A, Sharma N, Saleem RM (2001). Selective postoperative inhibition of gastrointestinal opioid receptors. N Engl J Med.

[17] Manzke T, Guenther U, Ponimaskin EG (2003). 5-HT4 (a) receptors avert opioid-induced breathing depression without loss of analgesia. Science.

[18] Romberg R, Olofsen E, Sarton E, Teppema L, Dahan A (2003). Pharmacodynamic effect of morphine-6-glucuronide versus morphine on hypoxic and hypercapnic breathing in healthy volunteers. Anesthesiology.

[19] Viscusi ER, Martin G, Hartrick CT, Singla N, Manvelian G (2005). EREM Study Group. Forty-eight hours of postoperative pain relief after total hip arthroplasty with a novel, extended-release epidural morphine formulation. Anesthesiology.

[20] Mather LE, Woodhouse A, Ward ME, Farr SJ, Rubsamen RA, Eltherington LG (1998). Pulmonary administration of aerosolised fentanyl: pharmacokinetic analysis of systemic delivery. Br J Clin Pharmacol.

[21] Fisher DM, Kellett N, Lenhardt R (2003). Pharmacokinetics of an implanted osmotic pump delivering sufentanil for the treatment of chronic pain. Anesthesiology.

[22] Salim K, Schneider U, Burstein S, Hoy L, Karst M (2005). Pain measurements and side effect profile of the novel cannabinoid ajulemic acid. Neuropharmacology.

[23] Kissin EY, Freitas CF, Kissin I (2005). The effects of intraarticular resiniferatoxin in experimental knee-joint arthritis. Anesth Analg.

[24] Strumper D, Durieux M E (2003). Topical Antidepressants: The New Local Anesthetics?. Reg Anesth Pain Med.

